# Improving maternal and neonatal outcomes in low-income communities in lagos state, Nigeria: an overview of the mamabase pilot program

**DOI:** 10.3389/fgwh.2026.1847263

**Published:** 2026-06-24

**Authors:** Bosede B. Afolabi, Onikepe O. Owolabi, Tope Olubodun, Fehintoluwa Aluko, Olufunmilola Owosho, Uche D. Azubuike, Olalekan Seun Olagunju, Chiamaka Uwalaka, Oluwafunke Iroko, Olajumoke Oke

**Affiliations:** 1 Maternal and Reproductive Health Research Collective, Lagos, Nigeria; 2 Faculty of Clinical Sciences, College of Medicine, University of Lagos, Lagos, Nigeria; 3 Department of Obstetrics and Gynaecology, Lagos University Teaching Hospital, Lagos, Nigeria; 4 Centre for Clinical Trials and Implementation Science (CCTRIS), College of Medicine, University of Lagos, Lagos, Nigeria; 5 Guttmacher Institute, New York, NY, United States

**Keywords:** antenatal care—maternal health—quality of care—health systems, community health workers (CHW), conditional cash transfer (CCT), maternal health, primary health care

## Abstract

Nigeria accounts for nearly one-fifth of global maternal deaths, with disproportionately high mortality among women living in low-income communities. Structural, financial, and sociocultural barriers continue to limit access to skilled maternal healthcare, in low-income settings in urban Lagos State. The MamaBase pilot program was implemented by the Maternal Reproductive Health Research Collective (MRH Collective) in 2024. The program was designed to address demand-side barriers to maternal health service utilization through a community-centered, data-driven approach, and was implemented across low-income communities in all 20 Local Government Areas of Lagos State, Nigeria. and. This prospective observational study describes the MamaBase pilot programme and presents early findings from its implementation. MamaBase employed a multicomponent approach that included community outreach and health education, active identification and enrollment of pregnant women by community health extension workers (CHEWs), linkage to nearby public primary healthcare facilities, and continuous follow-up throughout pregnancy and up to six weeks postpartum. Real-time data collection and monitoring of maternal health service utilization was done. Conditional cash incentives were provided to encourage early antenatal care (ANC) registration, completion of at least four ANC visits, and facility-based delivery, alongside targeted financial support for selected clinical needs. A total of 7,883 pregnant women were enrolled across the state. At enrollment, 47% of women were registered for ANC; this increased to 84% during program participation. Among women with available visit data, 58.5% attended four or more ANC visits. The majority of participants delivered in health facilities, most commonly public primary healthcare centers. The MamaBase pilot demonstrates the feasibility of a large-scale, community-centered maternal health intervention in low-income urban settings. Integrating demand-generation strategies, financial incentives, CHEW-led follow-up, and digital surveillance may strengthen maternal service utilization and reduce inequities in access to skilled care. Lessons from this pilot may inform the design and scale-up of similar community-based maternal health programs in other resource-constrained urban settings.

## 1 Introduction

Maternal mortality remains a major global public health challenge despite substantial improvements in maternal survival over the past two decades. Globally, maternal mortality reduced by 34% between 2000 and 2020 (1). Nevertheless, pregnancy and childbirth continue to pose significant mortality risk to women. These risks are most pronounced in low- and middle-income countries (LMICs), where women continue to face multiple barriers in seeking and receiving timely, quality maternal health care (2). In 2023, there were approximately 260,000 maternal deaths globally, and 92% occurred in LMICs, most of which could have been prevented (2). Delays in accessing skilled care, poor utilization of antenatal and delivery services, financial barriers, and weak health systems continue to contribute substantially to maternal deaths in many resource-constrained settings.

Nigeria, Africa's most populous country, contributes nearly 20% of global maternal deaths (3). According to modelled estimates by the World Health Organization (WHO), United Nations Children's Fund (UNICEF), United Nations Population Fund (UNFPA), and the World Bank, Nigeria's Maternal Mortality Ratio (MMR) was estimated at 993 maternal deaths per 100,000 live births in 2023 based, placing the country among those with a very high maternal mortality burden (4). Multiple interacting demand- and supply-side factors contribute to Nigeria's persistently high maternal mortality. On the demand side, women often delay seeking care because of limited awareness of pregnancy danger signs, sociocultural beliefs, low decision-making autonomy, and financial constraints (5–7). On the supply side, health system challenges – including distance to health facilities, transportation barriers, shortages of skilled health personnel, inadequate equipment and drugs, weak referral systems, and limited emergency obstetric and newborn care (EmONC) capacity—further impede access to timely and quality care (8–12). These challenges are particularly pronounced among women living in poverty (13).

Socioeconomic disadvantage significantly shapes maternal health outcomes in Nigeria. Women living in low-resource settings often have lower levels of education and health literacy and face substantial financial and geographic barriers to accessing health services. Many reside in informal settlements with limited infrastructure and may be unable to afford antenatal care (ANC) or facility-based delivery, increasing reliance on unregulated providers such as traditional birth attendants or home delivery (6). Although poverty is more prevalent in rural areas, approximately 42% of Nigeria's urban population lives in poverty, highlighting the vulnerability of low-income urban communities (14).

Lagos State, Nigeria's smallest yet most populous state, exemplifies these challenges. Despite being highly urbanized and economically significant, Lagos is characterized by considerable socioeconomic inequality, with approximately 65% of residents living in urban low-income communities (15). Maternal mortality in these low-income settings remain extremely high (16). Although public primary healthcare centers (PHCs) are widely available across Lagos State, structural, financial, and sociocultural barriers continue to limit utilization of maternal health services among women in low-income communities (17). This paradox of relatively high service availability alongside continued underutilization highlights the importance of demand-side interventions that address barriers to care-seeking and sustained engagement with maternal health services.

Evidence from the Iyaloju Initiative—a three-year maternal health quality improvement program in Lagos—highlighted several key factors influencing maternal health-seeking behavior (6). These included misinformation and cultural beliefs about pregnancy care, financial constraints, distance to facilities, partner influence, health worker attitudes, and long waiting times. The initiative also demonstrated the importance of community mobilization strategies—such as engaging community health workers, women's health champions, and local leaders—in strengthening trust between communities and healthcare providers and improving service uptake (6). Building on the lessons from the Iyaloju Initiative, the Maternal Reproductive Health Research Collective (MRH Collective) developed the MamaBase program to address demand-side barriers to maternal healthcare utilization in low-income urban communities in Lagos State.

MamaBase was designed as a community-centered intervention aimed at improving maternal and newborn outcomes by increasing the use of skilled maternal health services among vulnerable women. The program focuses on strengthening community-based identification and registration of pregnant women across all local government areas in Lagos State, linking them to nearby public primary healthcare facilities, and supporting them throughout the continuum of maternal care—from antenatal care to delivery, postnatal care, immunization, and family planning. In addition, MamaBase provides continuous follow-up throughout pregnancy and up to six weeks postpartum, incorporates conditional cash incentives to reduce financial barriers that discourage facility-based care, and utilizes a secure, interoperable digital data system to enable real-time monitoring of maternal health service utilization and support evidence-informed program implementation.

This article describes the development and pilot implementation of the MamaBase intervention in Lagos State in 2024 and presents preliminary findings on maternal health service utilization among enrolled participants. It also highlights key lessons learned during implementation and discusses implications for the design of community-based maternal health interventions in low-income urban settings.

## 2 Context: setting and population

The MamaBase pilot program was implemented in Lagos State, located in southwestern Nigeria. Lagos is the country's economic and commercial hub and one of the fastest-growing megacities in Africa, with an estimated population exceeding 16 million people and an annual population growth rate of approximately 3.7% (18, 19). Despite its highly urbanized and economically significant status, the state also contains numerous low-income urban communities and informal settlements. Rapid urbanization, high population density, and substantial socioeconomic inequality continue to place significant pressure on health systems and contribute to persistent gaps in access to and utilization of maternal health services, particularly among low-income populations (20). Administratively, Lagos State is divided into 20 Local Government Areas (LGAs) and 57 Local Council Development Areas (LCDAs), which are organized into six health districts responsible for coordinating public health service delivery. Across the state, approximately 310 functional primary healthcare facilities provide frontline services including antenatal care, delivery, immunization, and other essential maternal and child health services. The state has numerous private and public primary, secondary and tertiary facilities that provide maternal and child health services (21).

Despite the presence of a relatively extensive primary healthcare network, significant disparities persist in access to maternal health services across Lagos State. These disparities are particularly evident in low-income urban communities and informal settlements, where a large proportion of the population resides (6). Approximately 65% of people who migrate to Lagos live in densely populated urban low-income communities characterized by overcrowding, limited sanitation infrastructure, insecure housing, and restricted access to basic social services (15). These environmental and socioeconomic conditions contribute to poor health outcomes and limit the ability of women to access timely and appropriate maternal healthcare services.

Maternal health indicators in these settings remain concerning. For example, a household survey of nearly 4,000 households in two large informal settlements in Lagos reported maternal mortality ratio of 1,050 deaths per 100,000 live births, which is considerably higher than national estimates (16). Despite these challenges, Lagos has a relatively strong foundation of community-based health personnel who serve as critical links between communities and the formal health system, and can be leveraged to improve maternal health service utilization in underserved communities. Community Health Extension Workers (CHEWs) are an integral part of Nigeria's primary healthcare workforce and are typically deployed at the community level to provide basic preventive and promotive health services.

The MamaBase program was designed to operate within Lagos State urban health system environment. The pilot was implemented across low-income communities in all 20 LGAs of Lagos State.

## 3 Study design

This was a prospective observational study of the MamaBase pilot program, a community-based maternal health intervention implemented across low-income communities in Lagos State, Nigeria. Pregnant women enrolled in the program were followed from recruitment through pregnancy until six weeks postpartum using routinely collected programmatic and surveillance data, without the use of a control group.

## 4 Key programmatic and methodological components

**Figure 1:**
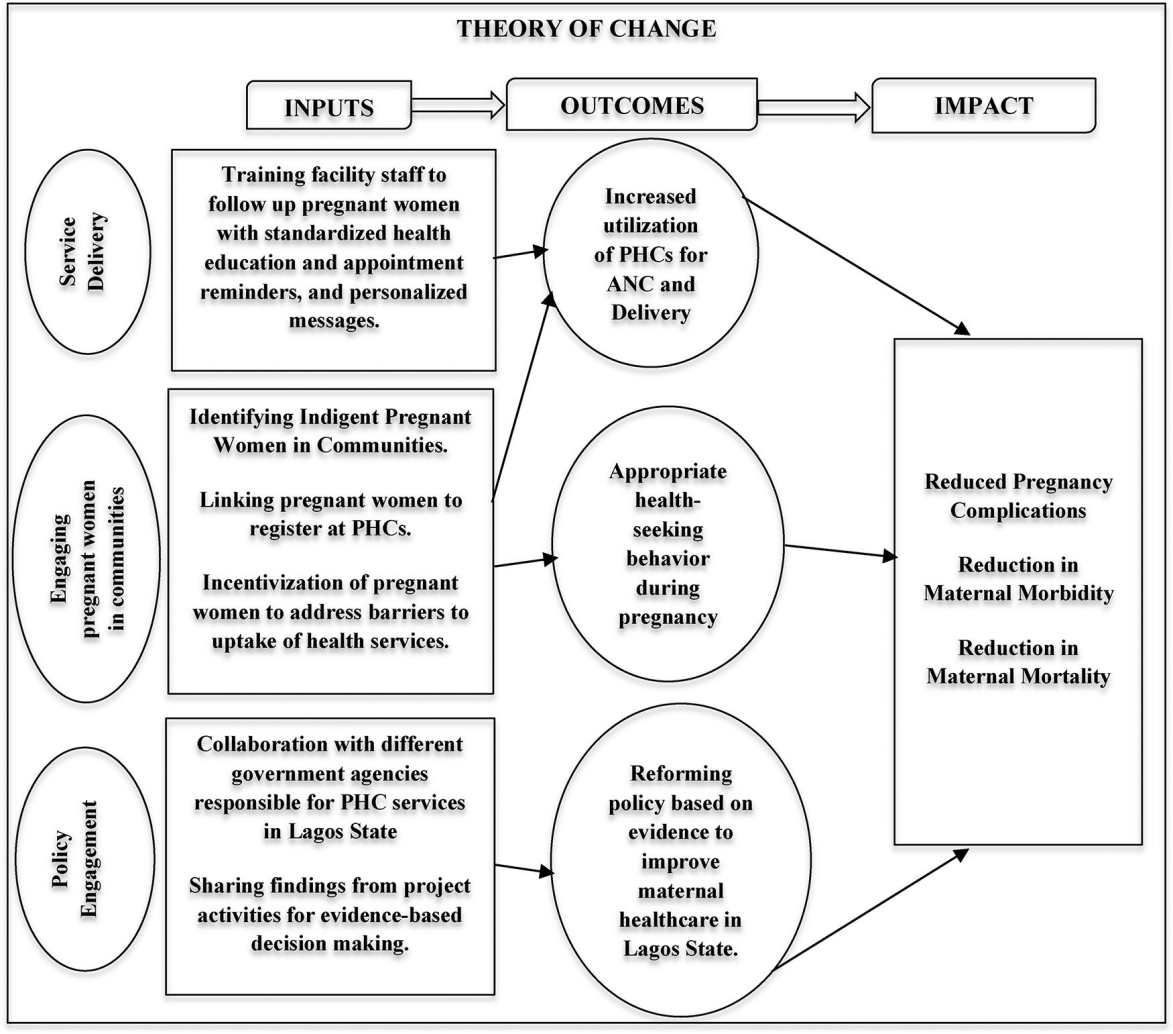
Mamabase project theory of change.

The overall conceptual framework underpinning the MamaBase intervention is presented in Figure 1. The theory of change illustrates how service delivery, community engagement, and policy engagement components were expected to contribute to improved maternal health service utilization and maternal outcomes.

### 4.1 Recruitment of women and eligibility criteria

Pregnant women were recruited through community mobilisation and outreaches and enrolled into the MamaBase program. Community mobilizers and CHEWS disseminated information about the programme widely. They identified eligible women within their communities and referred them for the recruitment exercise. The eligibility criteria for recruitment were any pregnant women with the telling sign of a protruding belly or positive result on a Rapid Pregnancy Test Kit. Stands were positioned within central community locations, in all LGAs, during the recruitment exercise. Simple health checks such as urinalysis, basic vital signs and blood pressure checks were conducted at the stands as an incentive for women to participate and be registered on the programme.

### 4.2 Sampling methodology

The study used purposive sampling. Low-income communities within each Local Government Area (LGA) were identified in collaboration with Community Health Extension Workers (CHEWs), community mobilisers, and local stakeholders based on characteristics such as high population density, poor housing, sanitation conditions, and socioeconomic disadvantage. In each LGA, community mobilisers and CHEWs recruited eligible pregnant women through community mobilisation activities and outreaches conducted within the selected communities. Across the 20 LGAs in Lagos State, a total of 7,883 pregnant women were enrolled into the programme and included in the analysis.

### 4.3 Personnel and training

CHEWS, community mobilisers and LGA co-ordinators were recruited for the program. A total of 120 CHEWs were recruited, and they were assigned across the 20 LGAs in Lagos State. The CHEWS and community mobilisers were supervised by 20 LGA coordinators, who were responsible for overseeing the activities in each LGA.

CHEWS were trained on Respectful Maternity Care, Human Research Ethics, participatory research approaches and data collection using Open Data Kits. LGA coordinators were also trained on data collection protocol for the MamaBase Program.

### 4.4 Linkages to care

Once registered in the programme, each pregnant woman was linked to a nearby primary healthcare center (PHC) through a CHEW, if they were not already registered for antenatal care. The CHEWs were responsible for ensuring that every woman is connected to the care and services needed for a healthy pregnancy and delivery. Linkage to care is a vital component of the program, ensuring that registered women receive the necessary antenatal care (ANC) and follow-up services during their pregnancy. The cost of registration and initial ANC screening tests were covered by the MamaBase project, for women who could not afford to pay.

### 4.5 Follow-up of pregnant women

Pregnant women received continuous follow-up throughout their pregnancy by CHEWs and the project monitoring team. In addition to weekly follow-up calls, the CHEWs were responsible for ensuring that the women in their care attended ANC visits, reported new symptoms, and provided feedback after each visit. The ANC follow-up tool containing details of the services received by pregnant women was administered after every ANC clinic, and other required information was obtained from facility records. This weekly ANC follow-up continued until delivery, after which the maternal and neonatal outcome tool was administered. The CHEWS also monitored the quality of care received by the women at their linked health facilities using the experience-of-care tool.

Weekly SMS reminders were provided on the importance of ANC attendance, recognizing danger signs in pregnancy, and the importance of birth preparedness. MamaBase program also provided follow-up through telephone calls, home and facility visits by trained CHEWs across the 20 Local Government Areas.

### 4.6 Community outreaches and health education

Community outreach activities served as a critical entry point for engaging pregnant women prior to the commencement of participant registration in each community. These outreach sessions were organized within the target communities and were primarily attended by pregnant women. The events provided an opportunity to introduce the program, build trust, and increase awareness of key maternal health issues.

During the sessions, trained facilitators delivered structured health education covering essential topics such as the identification of danger signs in pregnancy, appropriate nutrition during pregnancy, recognition of the onset of labour, and the importance of maintaining environmental hygiene. These outreach activities not only enhanced community knowledge but also created an enabling environment for subsequent enrollment and participation in the program.

### 4.7 Incentives and emergency support

To promote the uptake of essential maternal health services, the MamaBase program implemented a structured system of conditional cash incentives provided at key stages of pregnancy and childbirth. Incentives were designed to reduce financial barriers, reinforce positive health-seeking behavior, and encourage adherence to recommended antenatal and delivery practices. Women enrolled in the program were eligible to receive financial support at three points during their pregnancy journey.

First, upon registration in the program, each participant received ₦500 as an initial incentive to encourage early engagement with maternal health services. Second, women who completed four or more antenatal care (ANC) visits were awarded ₦1,500 ($1.02), thereby promoting continuity of care. Third, women who delivered in a recognized health facility received an additional ₦3,000 ($2.04), aimed at encouraging facility-based delivery and reducing the risks associated with home births. In total, each participant could receive up to ₦5,000 ($3.40) in conditional incentives over the course of her pregnancy.

In addition to routine incentives, the program also provided targeted emergency support for women facing specific clinical needs. This included assistance for women requiring caesarean section who encountered financial constraints at the time of delivery. Furthermore, emergency support was extended to women with Rhesus-negative blood type who required Rho(D) immune globulin (RhoGAM) to prevent alloimmunization. Because RhoGAM must be administered within 72 h after delivery and is often unaffordable for beneficiaries, the program helped ensure timely access to this life-saving intervention. Through these combined strategies, MamaBase aimed to improve maternal health outcomes by reducing financial barriers and responding promptly to urgent clinical needs.

### 4.8 Data collection, management, and surveillance

Data were collected longitudinally throughout implementation of the MamaBase programme using structured electronic questionnaires administered on REDCap by trained Community Health Extension Workers (CHEWs) and programme staff. The programme incorporated a structured surveillance system to monitor maternal health service utilization and outcomes among enrolled participants throughout pregnancy and the postpartum period. Routine follow-up was conducted weekly through telephone calls, home visits, and facility visits across the 20 LGAs of Lagos State. Data collection tools included the ANC Registration Form, ANC Follow-Up Form, Experience of Care Forms, Maternal and Neonatal Outcome Form, Verbal Autopsy Form, and Weekly ANC Attendance Records. At enrolment, information on sociodemographic characteristics, gestational age, reproductive history, and ANC registration status was collected. Follow-up data on ANC attendance, services received, women's experiences of care, delivery location, postnatal care attendance, maternal and neonatal outcomes, and incentives received were subsequently collected during pregnancy and within six weeks after delivery through household visits, phone calls, facility records, and programme registers. Information on maternal or neonatal deaths was documented using a verbal autopsy tool adapted from the World Health Organization verbal autopsy instrument. Data were collected in real time using mobile phone–based electronic forms, while access to the REDCap database was restricted to authorized study personnel to ensure confidentiality and data security.

### 4.9 Policy engagement

Policy engagement constituted a central component of the MamaBase project's implementation strategy, ensuring alignment with existing health system structures and fostering ownership among key stakeholders. Prior to the commencement of field activities, a series of stakeholder engagement meetings were convened with relevant government bodies and community leaders. These included the Lagos State Ministry of Health, the Lagos State Primary Health Care Board, Medical Officers of Health (MOHs), Ward Health Committee leaders, representatives from health training institutions, and community gatekeepers. These meetings were instrumental in introducing the project objectives, identifying context-specific needs, and securing institutional support for smooth implementation.

Throughout the implementation phase, the project team maintained active engagement with senior health personnel across multiple levels of care, including primary health centres (PHCs), general hospitals, Federal Medical Centres (FMCs), and tertiary health facilities. These interactions allowed for continuous communication, clarification of roles, and facilitation of collaboration necessary for registering pregnant women, monitoring service utilization, and responding to emerging operational challenges.

Following the completion of project activities, findings and results were disseminated through structured feedback sessions with district Permanent Secretaries, officials from the Lagos State Ministry of Health, and representatives of the Primary Health Care Board. Additionally, a press conference was organized to communicate key outcomes to the broader public, thereby promoting transparency and encouraging wider adoption of evidence-based maternal health interventions. Through this multi-level policy engagement approach, MamaBase ensured that its processes and outcomes were embedded within the existing health governance framework, enhancing both relevance and sustainability.

### 4.10 Data analysis

Data analysis was purely descriptive and was conducted using STATA version 17. Categorical variables were summarized using frequencies and percentages and presented in tables. No comparison group was used, and no inferential statistical comparison with baseline data was conducted.

## 5 Early findings From the mamabase pilot program

### 5.1 Distribution of registered pregnant women across the 20 LGAs in lagos state

Table 1 presents the number of pregnant women registered in each LGA. The distribution of participants varied across the LGAs, with the highest number of registrations occurring in Ikorodu (12.3%), followed by Ikeja (7.9%) and Alimosho (6.9%). Smaller proportions of women were registered in Ajeromi-Ifelodun (2.6%), Amuwo-Odofin (2.6%), and other LGAs with fewer than 500 registered participants.

**Table 1:** Sampling and pregnant women registered by local government area (LGA).

LGA	Frequency (*N* = 7,883)	Percentage
Agege	526	6.7
Ajeromi-Ifelodun	201	2.6
Alimosho	545	6.9
Amuwo-Odofin	203	2.6
Apapa	437	5.5
Badagry	365	4.6
Epe	335	4.3
Eti-Osa	335	4.3
Ibeju-Lekki	255	3.2
Ifako-Ijaiye	212	2.7
Ikeja	624	7.9
Ikorodu	969	12.3
Kosofe	485	6.2
Lagos island	280	3.6
Lagos Mainland	295	3.7
Mushin	384	4.9
Ojo	436	5.5
Oshodi-Isolo	367	4.7
Somolu	265	3.4
Surulere	364	4.6

### 5.2 Background characteristics of registered pregnant women

Table 2 shows the demographic characteristics of the 7,883 pregnant women registered in the program. The majority of the women (58.2%) were between the ages of 25–34 years. The vast majority were married (95.3%). Majority (65.9%) had completed secondary education while 15.4% had completed tertiary education. The majority of the women were self-employed (73.2%), followed by unemployed (17.1%). Regarding gestational age at the time of registration, most women were between 14 and 27 weeks (51.1%), with 18.1% ≤13 weeks and 30.8% in their third trimester. Concerning the number of living children, the majority of women (50.5%) had between 1 and 2 children, followed by those with 3–4 children (19.0%), while 29.2% had no children.

**Table 2:** Background characteristics of pregnant women registered.

Variables	Frequency (*N* = 7,883)	Percentage
Age group (years)		
15–24	2,042	25.9
25–34	4,588	58.2
35–44	1,221	15.5
≥45	32	0.4
Marital Status		
Divorced	16	0.2
Married	7,512	95.3
Separated	24	0.3
Single	331	4.2
Level of education		
Completed primary	891	11.3
Completed secondary	5,195	65.9
Completed tertiary	1,214	15.4
No formal education	371	4.7
Postgraduate	212	2.7
Employment Status		
Government employment	173	2.2
Private employment	591	7.5
Self-employed	5,771	73.2
Unemployed	1,348	17.1
Gestational age		
≤13 weeks	1,427	18.1
14–27 weeks	4,028	51.1
28–40 weeks	2,428	30.8
Number of living children		
No child	2,302	29.2
1–2 children	3,981	50.5
3–4 children	1,498	19.0
5+ children	102	1.3

### 5.3 Antenatal care (ANC) Status and visits

Table 3 outlines the ANC registration and number of ANC visits for the 7,883 women enrolled in the MamaBase program. The data provides insights into how many women were already registered for antenatal care at the time of their initial enrolment, the number of ANC visits women had attended during their pregnancy, and whether they sought care outside the designated healthcare facilities.

**Table 3:** Status of ANC at registration and ANC visits.

Variables	Frequency	Percentage
Initial ANC registration status	***N*** **=** **7,883**	
Not registered	4,170	52.9
Registered	3,713	47.1
ANC registration during MamaBase program	***N*** **=** **7,883**	
Not registered	1,261	16.0
Registered	6,622	84.0
ANC visits	***N*** **=** **5,504**	
1–3 visits	2,284	41.5
4+ visits	3,220	58.5
Sought care at another place	***N*** **=** **5,504**	
No	5,064	92.0
Yes	440	8.0

At the time of enrollment on the MamaBase programme, 47.1% of women (3,713 women) were already registered for ANC while 52.9% of the women (or 4,170 women) had not yet registered for ANC. Due to the efforts of the MamaBase programme, a total of 6,622 women (82.0%) out of the 7,888 women were registered for ANC.

When examining the number of ANC visits had by the 5,504 women who reported their ANC visit data, it was found that 58.5% (3,220 women) had attended 4 or more ANC visits. Forty-one percent (2,284 women) had attended between 1 and 3 ANC visits. A small portion of women, 8.0% (440 women), reported that they sought care at another location, outside of their assigned healthcare facility.

### 5.4 Delivery outcomes and place of delivery

Out of the 7,883 registered women, 94.8% had a live birth, while a small percentage experienced adverse outcome such as maternal death (0.1%), miscarriage (1.3%), neonatal death (0.4%), and stillbirth (1.3%). A total of 164 women (2.1%) were lost to follow-up.

Regarding the place of delivery, 51.7% of women delivered at a primary healthcare facility, 19.2% gave birth at private hospitals and 8.2% had home births (8.2%). Nine percent of women were delivered by Traditional birth attendants and 1.9% by quacks Table 4.

**Table 4:** Delivery outcome and place of delivery.

Variable	Frequency	Percentage
Delivery outcome	***N*** **=** **7,883**	
Live birth	7,473	94.8
Maternal death	9	0.1
Neonatal death	32	0.4
Miscarriage	102	1.3
Still birth	103	1.3
Lost to follow up	164	2.1
Place of delivery	***N*** **=** **7,473**	
Primary health care	3,864	51.7
Private hospital	1,434	19.2
Traditional birth attendant	680	9.1
Home	613	8.2
General hospital	598	8.0
Quack	142	1.9
Church/mission	142	1.9

### 5.5 Distribution of conditional cash incentives across LGAs

Table 5 presents the distribution of conditional cash incentives provided to women enrolled in the MamaBase programme across the 20 LGAs in Lagos State. A total of 2,926 women had their ANC registration and initial investigations paid for, 3,219 women received ₦1,500 for completing four or more ANC visits, and 5,838 women received ₦3,000 for delivering in a recognized health facility. Overall, 1,215 women met all eligibility criteria and received all incentives. The number of women receiving incentives varied across LGAs, with Ikorodu recording the highest number of women who received all incentives (201 women), followed by Agege (143 women) and Surulere (117 women).

**Table 5:** Distribution of conditional cash incentives received by women in the mamaBase program.

LGA	Number of women registered	Women whose ANC registration and initial investigations were paid for	Women receiving ₦1,500 for completing ≥4 ANC visits	Women receiving ₦3,000 for facility-based delivery	Women receiving all incentives
Agege	526	249	285	411	143
Ajeromi-Ifelodun	201	105	49	121	33
Alimosho	545	124	183	402	48
Amuwo-Odofin	203	76	95	155	20
Apapa	437	126	166	271	50
Badagry	365	91	80	238	25
Epe	335	154	106	234	65
Eti-Osa	335	156	134	251	65
Ibeju-Lekki	255	59	142	146	16
Ifako-Ijaiye	212	56	92	157	14
Ikeja	624	163	286	514	83
Ikorodu	969	469	458	704	201
Kosofe	485	144	222	346	51
Lagos island	280	117	127	260	55
Lagos Mainland	295	48	62	272	8
Mushin	384	174	126	233	54
Ojo	436	185	89	335	58
Oshodi-Isolo	367	134	153	293	65
Somolu	265	110	99	202	44
Surulere	364	186	265	293	117
**Total**	**7,883**	**2,926**	**3,219**	**5,838**	**1,215**

$1 = N1470 (Dollar to Naira exchange rate in 2024).

## 6 Discussion

This overview presents the design, implementation, and early findings from the MamaBase pilot program, a statewide, community-based maternal health intervention implemented across all 20 local government areas of Lagos State, Nigeria. The program combined community outreach and health education, active identification and registration of pregnant women, linkage to nearby public primary healthcare centres, continuous follow-up by trained CHEWs, conditional cash incentives tied to key service milestones, targeted emergency financial support, and real-time digital data collection and surveillance. Through these integrated components, MamaBase enrolled 7,883 pregnant women and increased antenatal care (ANC) registration from 47% at enrolment to 84% during program participation, with 58.5% of women attending four or more ANC visits. The majority of participants delivered in health facilities, most commonly public primary healthcare centres, and relatively low proportions of maternal death, neonatal death, stillbirth, and miscarriage were recorded among enrolled women, alongside a small loss to follow-up.

CHEWs play a significant role in the health systems in low- and middle-income countries, especially in low-income communities and among vulnerable women. The World Health Organization recognizes community health workers as a critical component of primary healthcare systems and has emphasized their role in extending essential health services to underserved populations, improving access to care, and addressing health workforce shortages (22). CHEWs and community mobilizers played a central role in the implementation of the MamaBase program and this may have contributed to the observed outcomes. The embedded presence of CHEWS within communities, the familiarity of CHEWs with local social norms, languages, and community leadership structures can help to bridge trust gaps between women and public health facilities, and address concerns related to provider attitudes, perceived quality of care, and previous negative experiences (22). This was leveraged upon in the MamaBase project. By identifying pregnant women within their communities and actively linking them to nearby health facilities for maternal care, CHEWs in the MamaBase program facilitated continuity of care and likely reduced loss to follow-up through sustained monitoring and follow-up. While the use of community health workers is well established, the deliberate and systematic approach to care linkage adopted in MamaBase represents a strengthened strategy for improving utilization of maternal health services.

The use of conditional cash incentives in the MamaBase program likely supported improved engagement with maternal health services by alleviating key financial barriers faced by poor and marginalized women. Small, milestone-based incentives tied to registration, completion of recommended antenatal care visits, and facility-based delivery likely encouraged early entry into care, sustained ANC attendance, and the use of skilled delivery services. These incentives could have helped offset direct and indirect costs associated with care-seeking, such as transportation, registration fees, and opportunity costs, which are well-documented deterrents to maternal health service utilization in low-income urban settings (6, 23, 24). In addition, the provision of targeted emergency financial support—particularly for women requiring caesarean sections or Rho(D) immune globulin—addressed critical gaps at moments of heightened clinical risk, where delays due to inability to pay can result in preventable morbidity or mortality. Importantly, the observed improvements in service uptake are unlikely to be attributable to financial incentives alone; rather, they appear to have operated synergistically with sustained health education, trust-based follow-up by Community Health Extension Workers, and facilitated linkages to care.

An important feature of the MamaBase program was the integration of real-time, community-generated data into routine implementation. Phone-based digital tools enabled timely tracking of service utilization, antenatal attendance, delivery locations, and maternal and neonatal outcomes, facilitating early identification of gaps such as delayed registration, missed visits, or loss to follow-up. This real-time surveillance strengthened accountability and responsiveness by making both community-level engagement and facility-level service utilization visible to program managers and implementers. The use of a secure, interoperable digital platform also created the potential for future integration with existing health information systems, while supporting more granular planning, resource allocation, and maternal health monitoring during programme implementation.

Findings from the MamaBase pilot project show a 37 percentage points increase in ANC registration alongside a high health facility delivery rate. Although our study results are descriptive and lack a comparison group, the observed increase in ANC uptake and facility-based delivery following enrolment is notable in the context of low-income urban communities with documented challenges in accessing skilled maternal health services. Other similar demand generating interventions have also reported improved uptake rates of maternal health services. A project in Southern Ethiopia that used community health education intervention delivered by women leaders to pregnant women led to significantly higher rates of ANC and health facility delivery uptake in women in the intervention group as compared to those in the control group (25). The Janani Suraksha Yojana (JSY) program in India, a conditional cash transfer scheme, to incentivise women to give birth in a health facility also showed an increase in ANC rates and facility births (26). In Ethiopia, leveraging community health workers in community engagement was found to be associated with improved utilisation of health service utilization, including ANC and skilled delivery among in rural communities (27). In Malawi, a CHEW led intervention including home visits to provide care to pregnant women and generate demand for maternal health services reported higher odds of receiving any ANC and at least four ANC visits in the intervention group (28).

Unlike our study findings, Nigeria's Subsidy Reinvestment and Empowerment Programme (SURE-P)—which combined behaviour change communication and conditional cash transfers with supply-side investments such as expanded health workforce capacity, improved infrastructure, and increased availability of essential drugs and consumables—reported increases in the monthly number of women completing four or more ANC visits, but did not demonstrate significant improvements in early ANC initiation or skilled delivery. Nevertheless, evidence from multiple well-implemented demand-generating interventions, as well as supply-focused programmes that integrate demand-side components, has shown increases in the uptake of maternal health services across diverse settings (25–29).

### 6.1 Strengths and limitations of the mamaBase pilot project

The MamaBase pilot demonstrates several notable strengths, including its large-scale implementation across all local government areas in Lagos State. First, the program was implemented at scale, reaching all 20 local government areas in Lagos State and enrolling nearly 8,000 pregnant women across diverse low-income urban communities. Second, MamaBase adopted a multicomponent, demand-side-focused design that addressed several interrelated barriers to maternal health service utilization simultaneously. Third, the integration of real-time digital data systems strengthened monitoring and follow-up by enabling timely tracking of service utilization and maternal and neonatal outcomes. In addition, early and sustained engagement with government stakeholders and alignment with existing health system structures enhanced the program's relevance, legitimacy, and potential for sustainability and policy uptake.

However, our findings should be interpreted in light of important limitations. The absence of a comparison group limits the ability to attribute observed improvements in service utilization and outcomes directly to the intervention. In addition, the lack of baseline comparability limits assessment of the extent to which observed outcomes differed from pre-intervention patterns. Selection bias may also have occurred due to the purposive nature of participant recruitment, as women who enrolled in the programme may have been more motivated to utilize maternal health services. Several indicators relied on self-reported data, which may be subject to recall or social desirability bias, and a small proportion of participants were lost to follow-up. As such, results from this overview reflect programmatic performance and feasibility rather than causal impact.

### 6.2 Implications for policy, practice, and scale-up

The findings from the MamaBase pilot have important implications for urban maternal health policy and practice in Nigeria. They underscore the need to integrate demand-side strategies—such as community engagement, care navigation, and targeted financial support—into routine primary healthcare programming to address persistent barriers to service utilization. The program also highlights the value of embedding community-based surveillance and digital data systems within existing health structures to improve monitoring, accountability, and responsive service delivery. In considering scale-up, the CHEW-led model represents a potentially cost-effective approach that leverages existing human resources and is well suited to dense urban settings. Lessons from the pilot are informing planned adaptations in the next iteration of MamaBase, including refinements to follow-up strategies, incentive delivery, and data integration to strengthen sustainability and system alignment.

### 6.3 Conclusion

In conclusion, the MamaBase pilot demonstrates the feasibility of implementing an integrated, community-centered maternal health intervention in low-income urban settings. By addressing demand-side barriers, strengthening linkages to primary healthcare facilities, and embedding real-time data systems, the program contributes to reducing inequities in access and improving the utilization of skilled maternal care. The MamaBase experience offers relevant insights for policymakers, program implementers, and global maternal health practitioners seeking scalable approaches to improve maternal and neonatal outcomes in resource-constrained urban contexts.

## Data Availability

The raw data supporting the conclusions of this article will be made available by the authors, without undue reservation.
